# *Akkermansia muciniphila* Protects Against Psychological Disorder-Induced Gut Microbiota-Mediated Colonic Mucosal Barrier Damage and Aggravation of Colitis

**DOI:** 10.3389/fcimb.2021.723856

**Published:** 2021-10-14

**Authors:** Tuo Chen, Rong Wang, Zhenglan Duan, Xiaomin Yuan, Yang Ding, Zeyu Feng, Fan Bu, Li Liu, Qiong Wang, Jinyong Zhou, Lei Zhu, Qing Ni, Guoping Shi, Yugen Chen

**Affiliations:** ^1^ Department of General Surgery, Affiliated Hospital of Yangzhou University, Yangzhou, China; ^2^ Department of Colorectal Surgery, Affiliated Hospital of Nanjing University of Chinese Medicine, Nanjing, China; ^3^ Glycomics and Glycan Bioengineering Research Center, College of Food Science and Technology, Nanjing Agricultural University, Nanjing, China; ^4^ Basic Pharmacology Laboratory, Affiliated Hospital of Nanjing University of Chinese Medicine, Nanjing, China; ^5^ Central Laboratory, Affiliated Hospital of Nanjing University of Chinese Medicine, Nanjing, China; ^6^ Collaborative Innovation Center for Cancer Medicine, Affiliated Hospital of Nanjing University of Chinese Medicine, Nanjing, China

**Keywords:** chronic restraint stress, colitis, colonic mucus, fecal microbiota transplantation, *Akkermansia muciniphila*

## Abstract

Psychological disorders are associated with increased risk of severe inflammatory bowel disease (IBD) by causing gut microbiota dysbiosis and colonic mucosal barrier damage. However, the interaction between chronic restraint stress (CRS), gut microbiota composition, and colonic mucus remains unclear. We demonstrated that mice under CRS conditions exhibited alterations in microbiota composition, disruption of colonic mucus, and aggravation of colitis. In addition, the abundance of *Akkermansia muciniphila* was significantly decreased in mice under CRS and UC patients with depression, and positively associated with the expression of MUC2. After antibiotic treatment, the recipient mice colonized with CRS microbiota showed barrier defects and severe colitis. Administration of *Akkermansia muciniphila* was found to restore colonic mucus and modify the gut microbiota. We confirm that CRS-mediated gut microbiota dysbiosis results in colonic mucosal barrier damage and aggravation of colitis. Our results suggest that *A. muciniphila* is expected to be a potential probiotic to protect and treat colonic mucus that is involved in IBD with psychological disorders.

## Introduction

Inflammatory bowel disease (IBD), comprising ulcerative colitis (UC) and Crohn’s disease (CD), has become a global burden with rapidly increasing morbidity in the past 20 years. Epidemiological surveys indicate that established prevalence populations of IBD occur in 1.5 million people in American and 2.2 million in Europe, and affected populations are gradually growing worldwide (Cosnes et al., 2011; Molodecky et al., 2012). Numerous studies have also demonstrated that a high frequency of psychological disorder, such as depression or anxiety, is observed in IBD patients. Depression affects an estimated over 25% persons with IBD, and two to three times higher than healthy individuals (Walker et al., 2008). It is noteworthy that depression triggers severity of IBD, which included lower quality of life, hospitalization, risk of surgery, and IBD flare (Panara et al., 2014; Mikocka-Walus et al., 2016; Kochar et al., 2018). However, mechanisms behind the role of psychological disorders or depression in the aggravation of IBD are underexplored.

Recent evidence suggests that the risk factors of depression in aggravating IBD are closely related to the brain-gut axis, which is considered a bidirectional link for interactions between the brain and the gut (Al Omran and Aziz, 2014). The gut is considered to be among the biological factors that significantly influence the function and structure of the brain. In turn, the brain modulates the gut microbiota and microenvironment. Previous data from animal models have demonstrated that the induction of depression is involved in the development of experimental colitis by immune system dysfunction and gut microbiota dysbiosis (Ghia et al., 2009; Gao et al., 2018). It has been suggested that gut microbiota, as an independent component, significantly modulates the brain-gut axis (Rhee et al., 2009). Therefore, screening specific gut microbes plays an important role in exploring a novel therapeutic option.

Colonic mucus has been recognized as the first physical barrier that effectively protects the colon against toxins and pathogenic microorganism invasion. It consists of two layers, an outer layer attached to the gut microbiota and an inner layer attached to the epithelium. Colonic mucus is produced and maintained by an extensively glycosylated mucin-2 (MUC2), which is secreted by goblet cells and renews the inner mucus layer approximately every hour (Johansson et al., 2011; Ambort et al., 2012). The damage of colonic mucus leads to the aggravation of colitis in mice and humans (Johansson et al., 2008; Fu et al., 2011; Johansson et al., 2014). Enhanced intestinal permeability and gut microbiota dysbiosis caused by chronic stress has also been reported to result in mucosal immune reactions and intestinal inflammation (Kiliaan et al., 1998; Wei et al., 2019). However, the relationship between the gut microbiota and its influence on colonic mucus within the brain-gut axis warrants further investigation.

Our results reveal that chronic restraint stress (CRS), a credible procedure for establishing a model of depression in mice, induced alterations in gut microbiota and dysfunction in the colonic mucosal barrier and prompted the development of experimental colitis. Furthermore, abundance of *Akkermansia muciniphila* (*A. muciniphila*) was significantly reduced in mice under CRS and UC patients with depression. Subsequently, gut microbiota transplantation experiments were performed in recipient mice to investigate the causal relationship between the gut microbiota and colonic mucosal barrier. *A. muciniphila* supplementation could relieve depression-like symptoms and aggravation of colitis in recipient mice.

## Materials and Methods

### Animals

Male C57BL/6N mice weighing 18–20 g were purchased from Charles River (Nanjing, China). All animals were group-housed (three to four mice per cage) in a specific pathogen-free (SPF) condition under 12 h light-dark cycle with diet and water *ad libitum*. The mice were adapted to the laboratory environment for 7 days prior to the start of the experiments. The experimental protocols were approved by the Ethics Committee for Animal Experiments of the Jiangsu Provincial Hospital of Traditional Chinese Medicine. All efforts were made to minimize the suffering and number of mice used during the experiments.

### Experiment 1: Chronic Restraint Stress and Dextran Sodium Sulfate–Induced Colitis Protocol

**Figure 1:**
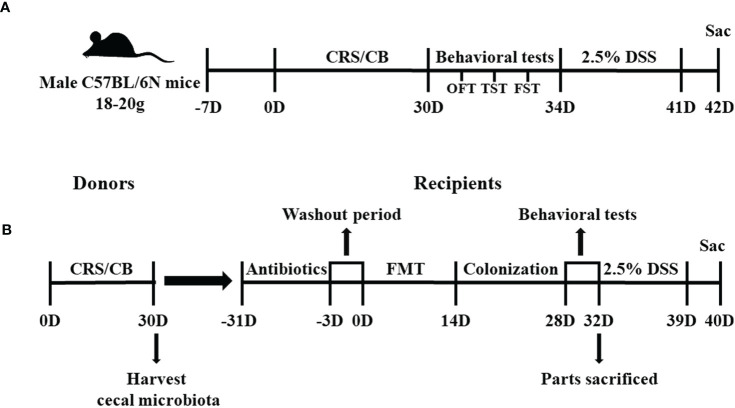
Study timelines of Experiments 1 and 2. **(A)** Graphical timeline of Experiment 1 including CRS and DSS-induced colitis. **(B)** Graphical timeline of Experiment 2 including antibiotic treatment and FMT. CRS, chronic restraint stress; DSS, dextran sodium sulfate; FMT, Fecal microbiota transplants.

In Experiment 1 (**Figure 1A**), 32 mice were randomly assigned to four groups: conventional breeding (CB; n = 8), CRS (n = 8), dextran sodium sulfate (DSS; n = 8), and DSS+CRS (n = 8). The CRS procedure was conducted in accordance with previously reported methods (Gao et al., 2018; Son et al., 2019). Briefly, mice in the CRS and DSS+CRS groups were limited to a 50 ml centrifuge tube with good ventilation for 3 h/day (9:00 a.m. to 12:00 a.m.) for 30 consecutive days, during which they were not allowed to move back or forth during the procedure. Behavioral tests were performed to assess depressive-related behaviors under CRS. Then, mice in DSS and DSS+CRS group were subjected to drink 2.5% (w/v) DSS (36–50 kDa, MP Biomedical) for 7 days. On day 8 after DSS administration, mice in all experimental groups were euthanized to assess the entire colon length and histological score. Two mice in the DSS+CRS group were removed from the experiment due to severe colitis that induced unexpected death during DSS administration.

### Experiment 2: Gut Microbiota Transplants and Colonization

In Experiment 2 (**Figure 1B**), 72 mice were randomly assigned to eight groups: donor CB (N = 10), donor CRS (N = 20), recipient-CB (RE-CB, n = 6), recipient-CRS (RE-CRS, N = 6), recipient CRS- *Akkermansia muciniphila* (RE-CRS-AKK, n = 6), recipient-CB-DSS (RE-CB-DSS, N = 8), recipient-CRS-DSS (RE-CRS-DSS, n = 8), recipient CRS*–Akkermansia muciniphila–*DSS (RE-CRS-AKK-DSS, N = 8). The mice in the donor CRS group were restrained in centrifuge tubes for 3 h daily for 30 consecutive days, as described above. All mice in the donor group were euthanized on day 31 to harvest the cecal microbial contents for fecal microbiota transplantation (FMT). Cecal contents from the donor group were mixed together in a 50 ml centrifuge tube based on the activity group (donor CB mice or donor CRS mice) and rapidly suspended in phosphate-buffered saline (PBS) with 0.1 g contents in 1 ml PBS. Cecal microbial suspensions were kept at −20°C and thawed overnight at 25°C prior to gavage. FMT was performed as previously described (Jimeno et al., 2018). Mice in the recipient group were given the following antibiotic cocktail *ad libitum* for 30 days to deplete all gut microbiota before FMT: ampicillin, neomycin, metronidazole (all at 1 g/ml), and vancomycin (0.5 g/ml). All antibiotics were obtained from Sigma-Aldrich. After 3 days washout period, 100 μl of frozen-and-thawed suspension was then administered by oral gavage into each recipient mouse for 14 days (Samuelson et al., 2017). In addition, two recipient groups treated with CRS suspension were orally administered with another 100 μl of *A. muciniphila* containing 1×10^8^ bacteria in parallel with groups given an equal volume of PBS as control (Routy et al., 2018). Behavioral tests were performed after gut microbiota colonization. At the end of the behavioral tests, some recipient mice were euthanized, and others received 2.5% DSS in drinking water for 7 days.

### Behavioral Tests

#### Open Field Test

The open field test (OFT) was conducted using an empty square apparatus (40 cm × 40 cm × 40 cm). Each mouse was gently placed in the corner of the open field arena, and its movements were recorded using a camera. The experimental arena was cleaned with 70% ethanol for each test. The total distance was analyzed using ANY-maze software to measure spontaneous activity.

#### Tail Suspension Test

The tail suspension test (TST) was performed to assess depression-like behavior and antidepressant efficacy, as described previously (Opal et al., 2014). Mice were individually suspended 50 cm from the floor by placing the tape 1 cm away from the tip of the tail. The test lasted for 6 min, and activity during the last 4 min was evaluated using ANY-maze software to quantify immobility time.

#### Forced Swim Test

The day after the TST, the mice were subjected to the forced swim test (FST) to assess depression-like behavior (Liu et al., 2016). Each mouse was individually placed in a glass cylinder (20 cm diameter, 40 cm height) containing water (23–25°C) for a duration of 6 min. Mice were kept immobile for the final 4 min and analyzed using the ANY-maze software. After the test, the mice were immediately placed in a clean and dry cage.

### *A. muciniphila* Culture

*A. muciniphila* strain Muc^T^ (DSM 22959) was kindly given by Prof. Li Liu (Glycomics and Glycan Bioengineering Research Center, College of Food Science and Technology, Nanjing Agricultural University, China). *A. muciniphila* was grown in brain heart infusion broth for 48 h at 37°C in an anaerobic incubator (Sheldon Manufacturing, USA) containing a N_2_/CO_2_/H_2_ (86:7:7) gas phase (Derrien et al., 2004; Shin et al., 2019). The final suspensions of *A. muciniphila* with 1×10^9^ colony-forming units (CFU)/ml were determined by measuring the optical density in PBS under 600 nm (Routy et al., 2018).

### Patient Characteristics

We obtained 35 human fecal samples from patients with active UC from the Jiangsu Province Hospital of Chinese Medicine. The study was performed according to the principles of the Helsinki Declaration, and approval was obtained from the Institutional Review Board of Jiangsu Provincial Hospital of Traditional Chinese Medicine, China on October 12, 2018 (approval code 2018NL-093-02). Depression was assessed using the Patient Health questionnaire-9 (PHQ-9) scales (Kroenke et al., 2010). Patients with scores ≥10 were defined as moderate to severe depression and included (Kroenke et al., 2001). The exclusion criteria were as follows: systemic infectious diseases, autoimmune diseases, gastrointestinal surgery, and history of other mental disorders. The patient characteristics are shown in **Supplementary Table 1**.

### *A. muciniphila* Quantification

The abundance of *A. muciniphila* in 35 fecal samples from patients with active UC was quantified using quantitative real-time PCR analysis. Bacterial DNA isolation was conducted using the Stool DNA Isolation Kit (TIANGEN, China) according to the manufacturer’s instructions. The quantity and purity of the extracted DNA were assessed using a NanoDrop 2000 spectrophotometer (Thermo Scientific, USA). Previously reported gene-targeted primers were used for the detection of *A. muciniphila* species (AM1-F: 5’-CAGCACGTGAAGGTGGGGAC-3’; AM2-R: 5’-CCTTGCGGTTGGCTTCAGAT -3’) (Collado et al., 2007). The *A. muciniphila* primers were purchased from Invitrogen (USA). Amplification reactions were performed in a total volume of 20 μl, including 2 μl of DNA template, 10 μl SYBR Green PCR Master Mix 2× (TaKaRa, China), and 0.5 μl of each primer and probe. The ABI 7500 real-time PCR system (Applied Biosystems, USA) was used to conduct the assays. The thermal profiles were constructed based on this protocol. In summary, the samples were held at 55°C for 2 min and 95°C for 10 min, followed by 40 cycles of 95°C for 15 s and annealing at 60°C for 1 min. Standard curves were obtained by comparing the cycle threshold (CT) with 10-fold serial dilutions of standard DNA templates from the culture of *A. muciniphila* strain Muc^T^ (DSM 22959) as previously reported (Song et al., 2004). Each analysis was run in duplicate on the same plate. The assay results were expressed as Log10 CFU/g of fecal stool.

### RNA Sequencing and Function Analysis

Total RNA was extracted from 12 frozen colonic tissue samples from mice [three tissues per group × four groups (CB, CRS, DSS, and DSS+CRS)] using a total RNA Extractor kit (Sangon Biotech, China) according to the manufacturer’s protocol. The quality and quantity of the isolated RNA samples were assessed using a Qubit 2.0 Fluorometer (Thermo Scientific). Libraries were established using the VAHTS mRNA-seq V2 Library Prep Kit for Illumina (Vazyme Biotech, China) according to the manufacturer’s instructions. Subsequently, libraries were sequenced on the Illumina Hiseq TM 2500 platform, generating an average of 150 bp paired-end reads (Reuter et al., 2015). To evaluate differentially expressed transcripts (DEGs) between groups, we annotated and quantified the transcripts using DESeq (Anders and Huber, 2010). Finally, the absolute value of log2 fold change was calculated to extract the DEGs (greater than 0.50 and p < 0.05). Volcano maps or heat maps were used to visualize the differential genes. To evaluate the function of DEGs, we performed Kyoto Encyclopedia of Genes and Genomes (KEGG) pathway enrichment analysis using KOBAS 3.0. DAVID (v6.8) was used to perform the gene ontology (GO) functions enriched based on the DEGs. Pathways containing more than two differentially expressed genes were screened. The RNA-sequence read data were deposited in the Sequence Read Archive (SRA) at the NCBI database (accession number: PRJNA758283).

### Quantitative Polymerase Chain Reaction

Quantitative polymerase chain reaction (qPCR) was used to confirm the differentially expressed genes of MUC2 in Experiments 1 and 2. Total RNA from colonic samples was isolated using TRIzoI Reagent (Invitrogen). After reverse transcription of RNA into cDNA using the Prime Script RT reagent Kit (TaKaRa, China), qPCR was conducted using the ABI 7500 real-time PCR system (Applied Biosystems) with SYBR Green PCR Master Mix 2× (TaKaRa, China). Gene changes were calculated using the 2^−ΔΔCt^ method by normalizing to glyceraldehyde 3-phosphate dehydrogenase (GAPDH) (Merrick et al., 2012). Primer details are shown in **Supplementary Table 2**.

### Histology and Immunohistochemistry

Colons were removed from the mice, flushed with PBS, fixed in 10% formaldehyde overnight at room temperature, and embedded in paraffin. The specimens were subsequently sectioned at 5 μm thick and stained with hematoxylin and eosin (H&E). Three slides in H&E staining of each colon were selected and analyzed by a blinded pathologist. Histopathological scores were assessed based on a previously described method (Sann et al., 2013).

Immunohistochemistry analysis was carried out as follows: the prepared sections were treated with primary antibodies using horseradish peroxidase*-*conjugated goat* *anti-rabbit* *IgG (Servicebio, China) overnight at 4°C. After washing in PBS, the slices were treated with the secondary antibody rabbit anti-MUC2 (Servicebio, China) for 50 min and then stained with diaminobenzidine. The expression of MUC2 were expressed as the number of positive cells per villus using ImageJ software.

### Periodic Acid-Schiff and Alcian Blue Staining

After deparaffinization and rehydration, the sections were stained with periodic acid-Schiff and Alcian blue (PAS/AB). The goblet cells are blue. The number of goblet cells was counted using Image J software and expressed as positive cells per villus.

### Gut Microbiota Analysis

Microbial DNA from cecal specimens was extracted using a DNA extraction kit (Tiangen, China) from samples of approximately 100 mg, following the manufacturer’s instructions. The 16S rRNA gene sequencing was conducted using Novogene. Briefly, the V3–V4 region of the microbial 16S rRNA gene was amplified with specific primers 341F (5’-ACTCCTACGGGAGGCAGCAG-3’) and 806R (5’-GGACTACHVGGGTWTCTAAT-3’). After the PCR product was purified, amplicon libraries were generated using the Illumina TruSeq DNA PCR-Free Library Preparation Kit (Illumina, USA) and then sequenced on the Illumina HiSeq platform for paired-end 250 bp raw reads. Collected raw data from the gut microbiota were uploaded to the NCBI database with the accession number PRJNA756202. To obtain high-quality data, reads containing more than 10% unknown nucleotides and less than 80% of bases with quality (Q-value) >20 were discarded (Ding et al., 2020). Subsequently, operational taxonomic units (OTUs) were clustered using UPARSE (v7.1) with a 0.97 threshold (Edgar, 2013). Each OTU was annotated using taxonomic information analysis according to the RDP database (Wang et al., 2007). All the data were analyzed by I-Sanger platform (http://www.i-sanger.com). We used the Shannon index as a metric to analyze differences in alpha diversity between the groups. Additionally, principal coordinate analysis (PCoA) was performed using unweighted UniFrac distances at the OTU level, and an analysis of similarity test (ANOSIM) was performed to assess significant differences in the bacterial community composition. Spearman correlation was used to analyze the relationships between the relative abundance of *A. muciniphila* and stress-associated gene counts, and the results were visualized using Cytoscape (v3.5.1).

### Statistical Analysis

All results are shown as mean ± SEM. Data were analyzed by one*-*way analysis of variance (ANOVA) with* *Tukey–Kramer test between two groups and two-way ANOVA with Bonferroni’s *post-hoc* test for multiple comparisons. Differences were considered statistically significant at p < 0.05.

## Results

The study timeline of the two independent experiments is depicted in **Figure 1**.

### Experiment 1: CRS Caused Adverse Psychological Effects, Colitis Aggravation, and Microbiota Dysbiosis

#### CRS Induced Depressive-Like Behaviors and Severe Colitis

**Figure 2:**
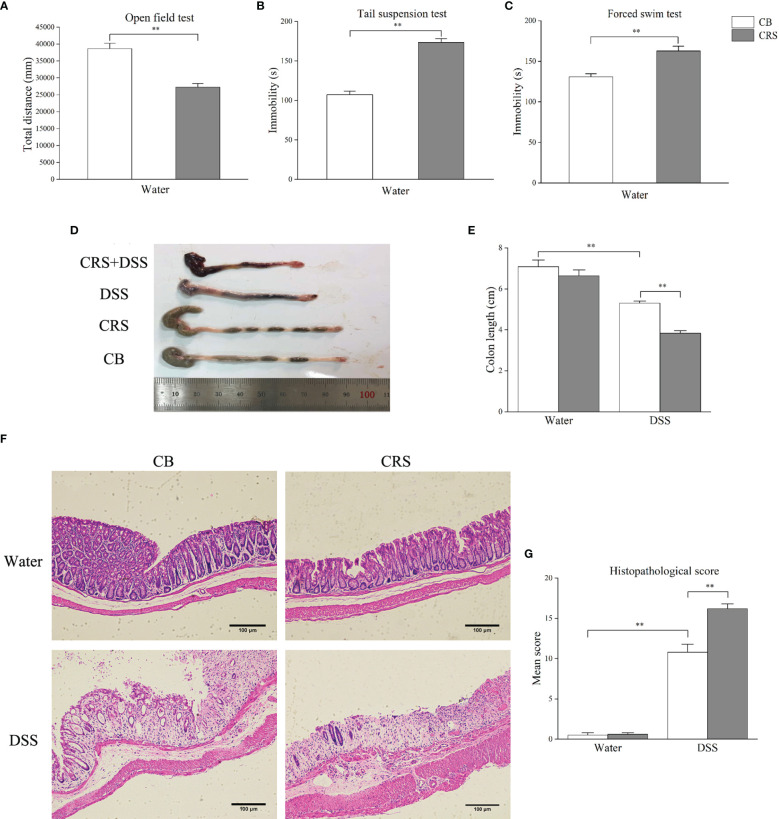
CRS aggravated DSS-induced colon injury. **(A–C)** Behavioral tests were performed before DSS, including OFT, TST, FST (n = 8). **(D)** Representative pictures of colon were selected between groups; **(E)** Colon length was recorded from each group (n = 6–8). **(F)** Representative pathological pictures of colonic injury were selected between groups (magnification ×40, scale bar: 100 μm). **(G)** Colonic injury score was assessed by histopathological score based on H&E staining (n = 5). *p < 0.05, **p < 0.01.

The CRS animal model exhibited depression-like behavior prior to DSS administration. Compared with the CB group, CRS mice showed significantly less movement distance in the OFT (**Figure 2A**). In addition, increased immobility and reduced swimming durations were observed in mice under CRS conditions compared to those in the CB group (**Figures 2B, C**). Following DSS exposure for 7 days, CRS mice developed severe colitis characterized by bloody diarrhea, severe infiltration of inflammatory cells, extensive villus injury of the bowel wall, and hyperplasia of reactive epithelial cells. Colon length was shorter in the DSS+CRS group than in the DSS group (**Figures 2D, E**). In addition, the histopathological scores were higher in the DSS+CRS group than in the DSS group (**Figures 2F, G**).

#### *A. muciniphila* Abundance Was Reduced in CRS Mice

**Figure 3:**
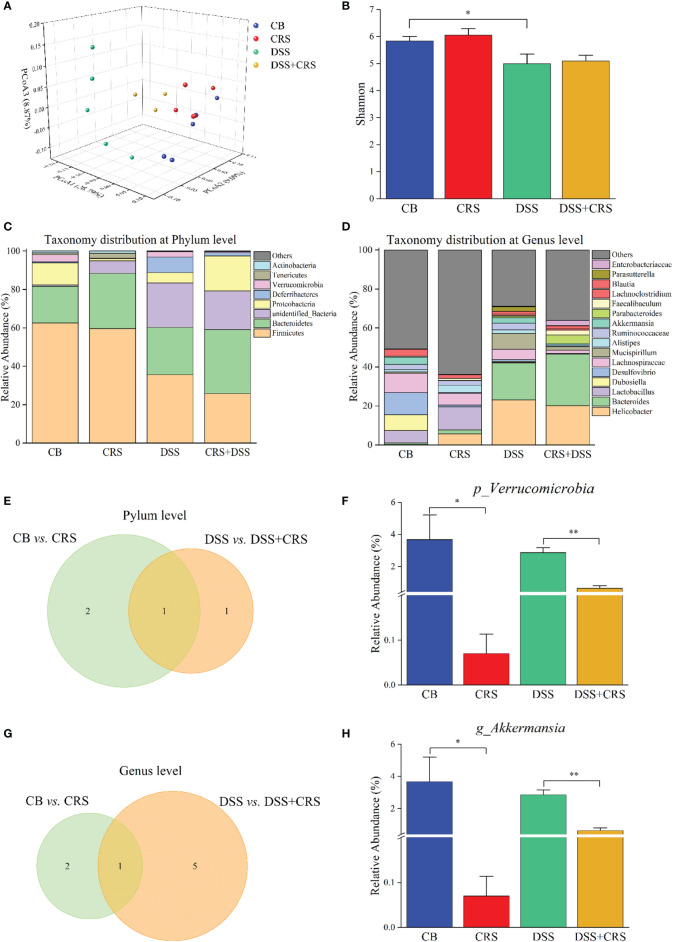
Reduction of *A. muciniphila* was observed in CRS mice. **(A)** PCoA according to unweighted Unifrac presented a clear site-specific clustering (n = 3–5). **(B)** Shannon index values among four experimental groups. **(C)** Microbiota composition at the phylum level. **(D)** Microbiota composition at the genus level. **(E)** Venn diagram showing only one differentially abundant species at the phylum level altered under CRS. **(F)** Relative abundance of *Verrucomicrobia* significantly decreased between groups. **(G)** Venn diagram showing only one differentially abundant species at the genus level altered under CRS. **(H)** Relative abundance of *A muciniphil*a had significantly decreased between different groups. *p < 0.05, **p < 0.01.

We utilized 16S rRNA sequencing to quantify and characterize the gut microbiota of mice under CRS conditions. We observed a decrease in the Shannon index in the DSS group compared to that in the CB group (p < 0.05), whereas no difference in alpha diversity was observed from the comparisons of CB *versus* CRS, and DSS *versus* DSS+CRS, respectively (**Figure 3B**). Unweighted UniFrac PCoA analysis indicated a distinct difference in beta-diversity at the OTU level among the four groups (**Figure 3A**). In contrast to the CB group, the microbiomes of mice in the CRS group exhibited significant changes based on the ANOSIM values (R = 0.761, p = 0.01). However, when comparing the DSS group with the DSS+CRS group, we found no differences (R = 0.323, p = 0.13). Next, we analyzed the microbiota community structure of each group. At the phylum and genus levels, the composition of microbiota showed dramatic changes in the CRS group or DSS+CRS group (**Figures 3C, D**). At the phylum level, three and two dominant phyla were identified from the comparisons of CB *versus* CRS, and DSS *versus* DSS+CRS, respectively (**Figure 3E**). In comparison to the CB group, the relative abundance of *Proteobacteria* and *Verrucomicrobia* was decreased and that of *Melainabacteria* was increased in the CRS group (**Figures S1A, B**). The relative abundance of *Proteobacteria* in the CRS+DSS group was higher than that in the DSS group, while the relative abundance of *Verrucomicrobia* was decreased. The relative abundance of *Verrucomicrobia* was significantly decreased in both comparisons at the phylum level (**Figure 3F**). At the genus level, the Venn diagram revealed three and six alternations of dominant genera from the comparisons of CB *versus* CRS, and DSS *versus* DSS+CRS, respectively (**Figure 3G**). When compared with the CB group, the relative abundance of *Odoribacter* was increased and that of *Akkermansia* and *Desulfovibrio* was decreased in the CRS group (**Figures S1C, D**). Moreover, there was a marked increase in the proportion of *Erysipelatoclostridium*, *Enterococcus*, and *Parabacteroides* and a decrease in *Romboutsia*, *Dubosiella*, and *Akkermansia* in the DSS+CRS group compared to those observed in the DSS group (**Figures S1E–I**). The mice subjected to CRS exhibited decreased *Akkermansia* abundance in the two comparisons at the genus level (**Figure 3H**).

#### Prevalence of *A. muciniphil*a in UC Patients With Depression

**Figure 4:**
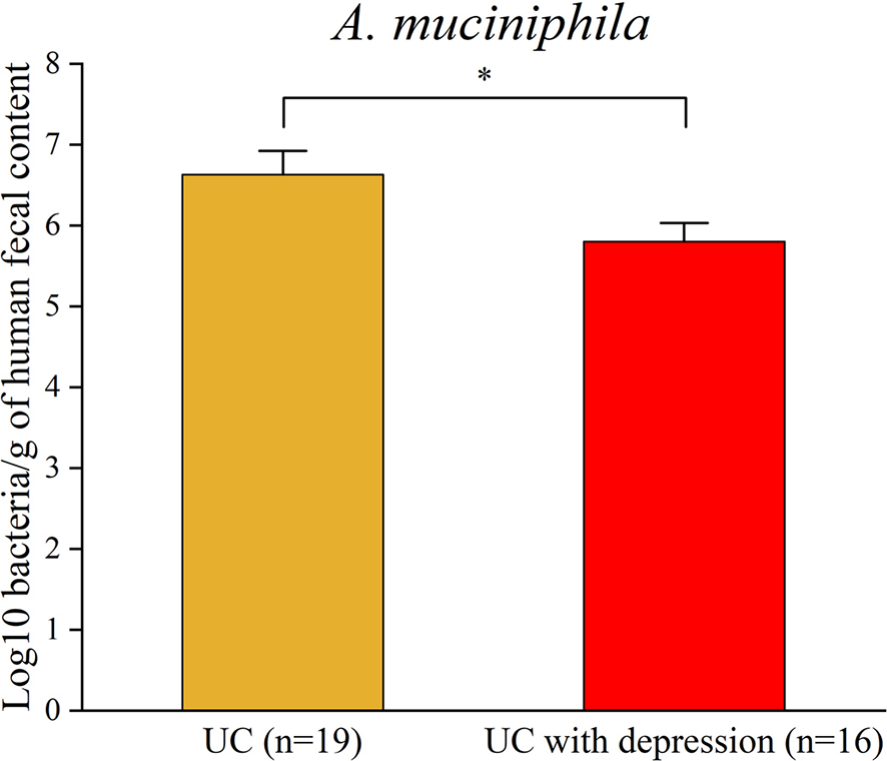
Real-time PCR analysis validated the reduction in *A. muciniphila* in UC patients with depression. *p < 0.05.

To assess prevalence of *A. muciniphil*a in UC patients with depression, a total of 35 fecal samples were collected from 16 UC patients with depression and 19 UC patients as control. Real-time PCR data further affirmed that the abundance of *A. muciniphila* was reduced in UC patients with depression (**Figure 4**). Above all, these results demonstrated that a lower abundance of *A. muciniphil*a under CRS might be associated with aggravation of colitis.

#### Interactions Between Differentially Expressed Genes Regulated by CRS and *A. muciniphila* in Mice

**Figure 5:**
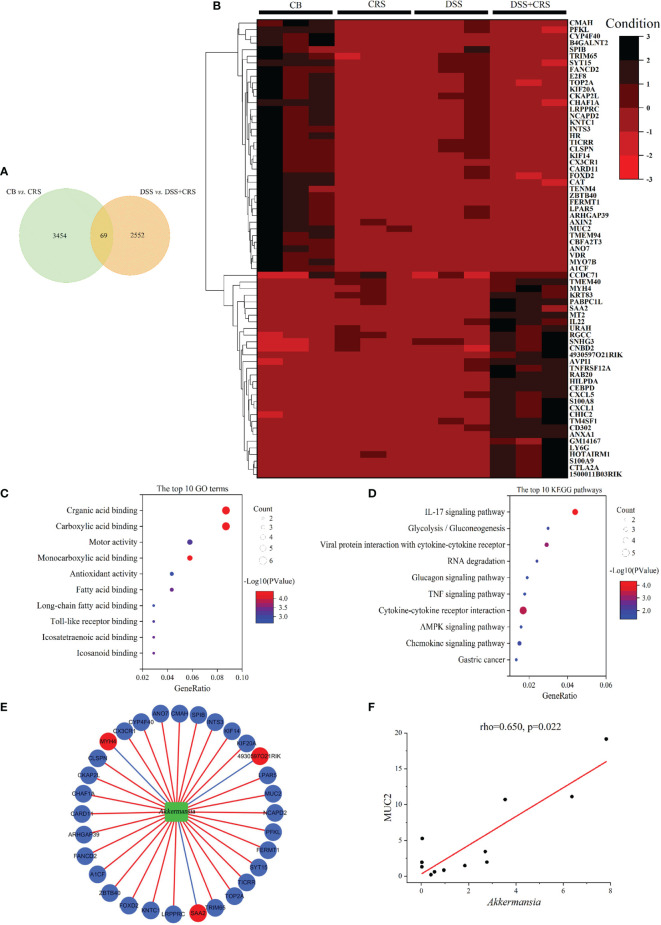
DEGs under CRS were analyzed by high-throughput RNA-Seq. **(A)** Venn diagram showing 69 DEGs changed under CRS from the two comparisons. **(B)** Heat-map presented 69 DEG in the two comparisons (n = 3). **(C)** The 10 most disordered GO terms were identified from the 69 DEG in the two comparisons. **(D)** The top 10 KEGG pathways were identified from the 69 DEG in the two comparisons; **(E)** Network visualized the correlations between *A. muciniphila* and genes. Green node represented *A muciniphila*. Red and Blue nodes represented the raising and lowering of DEGs. Blue lines represented negative correlation, and red lines represented positive correlation; **(F)** Scatterplot showing that MUC2 was positively correlated with *A. muciniphila*.

To determine how CRS exerted a harmful effect in DSS-induced colitis, we conducted DEG profiles in the colonic tissue of mice from the comparisons of CB *versus* CRS, and DSS *versus* DSS+CRS. Hence, 3,523 and 2,621 DEGs were identified respectively (**Figures S2A, D**). Notably, 69 DEGs were significantly altered in the two comparisons (**Figure 5A**). Expression levels of these 69 DEGs are shown as a heat map (**Figure 5B**). The 15 upregulated and downregulated genes most related to the two comparisons are presented in **Supplementary Table 3** and **Supplementary Table 4**. GO and KEGG analyses have been widely used to explore biological functions and large-scale pathway information based on the high-throughput sequencing data. When comparing the CB with CRS mice, the 10 most significant GO terms revealed that CRS mainly controlled the structural constituents of ribosomes, transcriptional coregulator activity, and enzyme activator activity (**Figure S2B**). KEGG pathway analysis showed that CRS mostly regulated Alzheimer’s, Parkinson’s, and Huntington’s diseases (**Figure S2C**). When comparing the DSS with DSS+CRS mice, the 10 most significant GO terms indicated that CRS was related to receptor ligand, cytokine, and cytokine receptor activity (**Figure S2E**). Pathway analysis revealed that the 10 most enriched pathways included the IL-17 signaling pathway, TNF signaling pathway, and Th17 cell differentiation pathway (**Figure S2F**).

Subsequently, 69 DEGs were screened from the above two comparisons and subjected to GO and KEGG pathway analyses. GO analysis showed that the identified DEGs were associated with fatty acid binding, long-chain fatty acid binding, and Toll-like receptor binding (**Figure 5C**). KEGG pathway analysis revealed that the DEGs were related to the IL-17, TNF, and AMPK signaling pathways (**Figure 5D**).

To verify the relationship between host genes and altered microbial communities and to identify their potential roles in worsening the severity of colitis, we performed a correlation analysis between 69 DEGs regulated by CRS and *A. muciniphila*. The correlation analysis revealed that 34 DEGs were significantly correlated with *A. muciniphila* (**Figure 5E**). Notably, MUC2 expression was positively correlated with *A. muciniphila* (**Figure 5F**).

#### CRS Inhibited Mucus Production and Goblet Cell Numbers in the Colon

**Figure 6:**
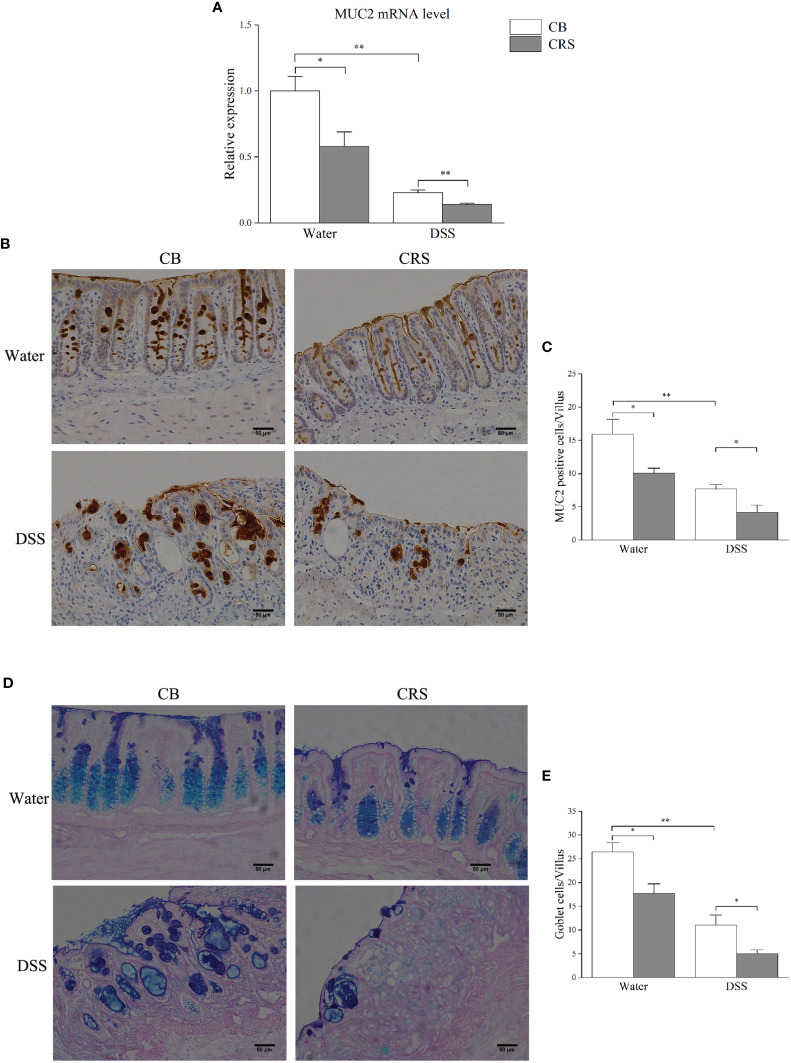
Chronic restraint stress prompted colonic mucosal barrier damage. **(A)** Relative expression of MUC2 was validated by qPCR. **(B)** Representative pictures shown immunostaining of MUC2 in colon (magnification ×200, scale bar: 50 μm). **(C)** The numbers of MUC2-positive cells in each villus (n = 4–5); **(D)** Representative pictures showing that colonic specimens stained with PAS/AB (magnification ×200, scale bar: 50 μm); **(E)** Numbers of goblet cells in each villus (n = 4–5). *p < 0.05, **p < 0.01.

qRT-PCR was performed to validate the expression of MUC2, which was selected from candidate DEGs, and correlation analysis. Our results demonstrate that CRS decreased MUC2 expression (**Figure 6A**), and MUC2-positive cells in each villus were significantly decreased in mice under CRS (**Figures 6B, C**). Due to mucin produced by goblet cells, we next examined the colon in mice from different groups and counted the number of goblet cells by PAS/AB staining. Our results revealed that the number of goblet cells was significantly decreased in all CRS mice compared to that in non-CRS mice (**Figures 6D, E**).

### Experiment 2. *A. muciniphila* Supplementation Prevented Mucosal Barrier Defects and Aggravation of Colitis

#### *A. muciniphila* Supplementation Alleviated Depression-Like Behaviors and Aggravated Colitis in Recipient Mice

**Figure 7:**
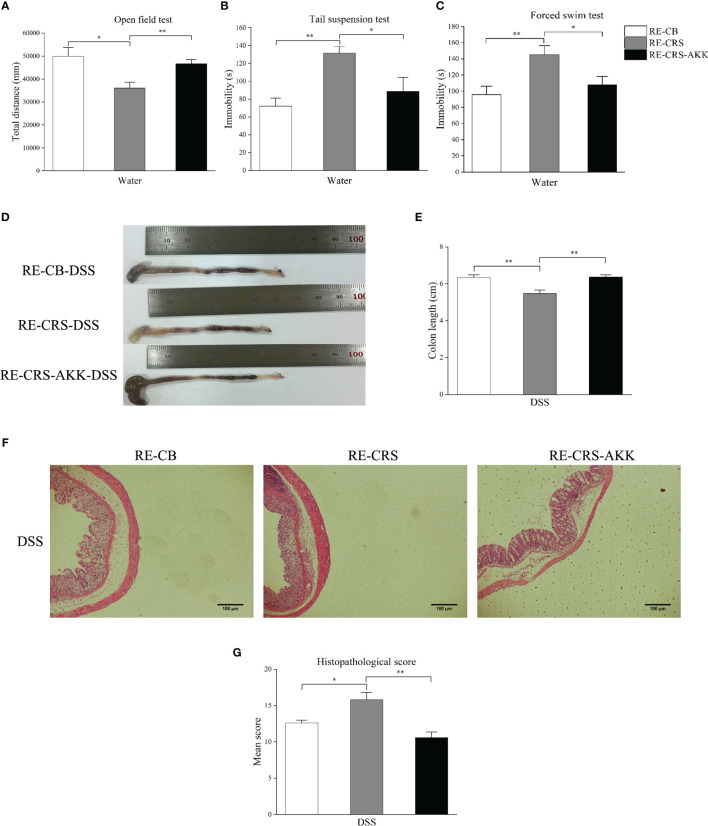
*A. muciniphila* supplementation alleviated depression-like behavior and colitis aggravation in recipient mice. **(A–C)** Depression-like behavior in recipient mice was alleviated by oral *A. muciniphila* supplementation by behavioral tests (n = 6). **(D)** Representative colon photographs from mice receiving DSS treatment. **(E)** Colon length recorded at time of sacrifice (n = 8). **(F)** Representative pathological photographs of colonic injury in mice receiving DSS treatment (magnification ×40, scale bar: 100 μm). **(G)** Colonic injury score assessed by histopathological score based on H&E staining in recipient mice (n = 4–5). *p < 0.05, **p < 0.01.

To further evaluate the role of CRS-driven dysbiosis in the aggravation of colitis associated with *A. muciniphila*, we performed FMT in recipient mice. Compared to the recipient mice colonized with the CB microbiota, the recipient mice colonized with the CRS microbiota showed increased depression-like behaviors in the OFT, TST, and FST. When compared with recipient mice colonized with CRS microbiota, *A. muciniphila* supplementation alleviated depression-like behaviors, including decreased immobility time in the TST and FST, and increasing movement distance in the OFT (**Figures 7A** **–C**). After DSS administration for 7 days, significant severe colitis was observed in recipient mice colonized with CRS microbiota compared to the CB microbiota. Conversely, *A. muciniphila* supplementation increased colon length and reduced the histopathological scores compared to the recipient mice colonized with CRS microbiota (**Figures 7D** **–G**).

#### *A. muciniphila* Supplementation Inhibited Colonic Mucosal Barrier Damage in Recipient Mice

**Figure 8:**
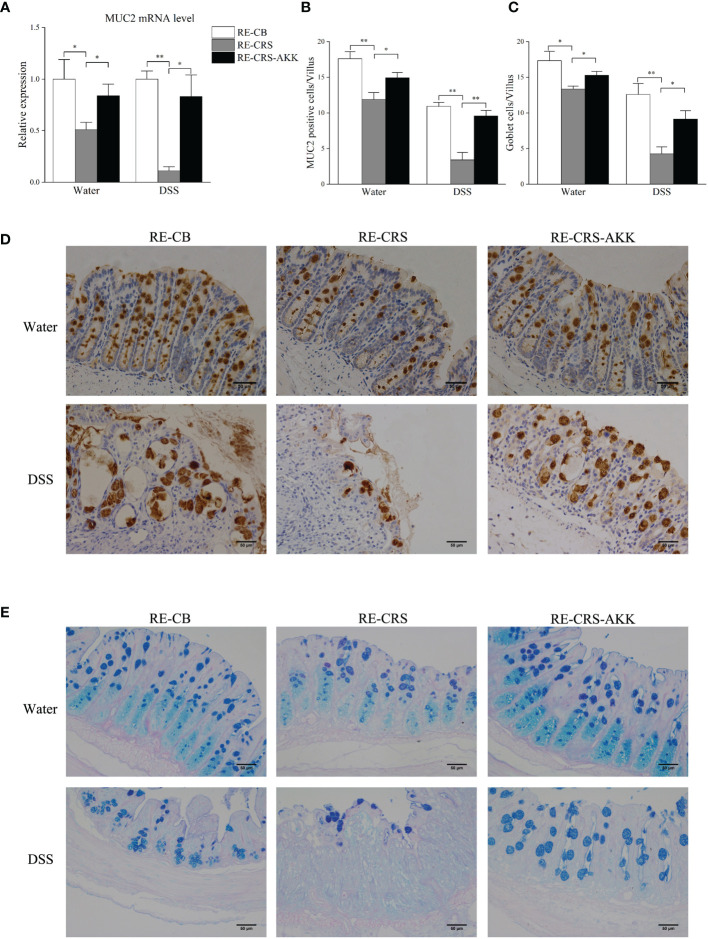
*A. muciniphila* supplementation protected colonic mucosal defects in recipient mice. **(A)** *A. muciniphila* supplementation affected relative expression of MUC2 in mice. **(B)** Numbers of MUC2-positive cells in each villus (n = 4–5). **(C)** Numbers of goblet cells in each villus (n = 4–5). **(D)** Representative photographs of colonic MUC2 immunostaining (magnification ×200, scale bar: 50 μm). **(E)** Representative photographs of colonic specimens stained with PAS/AB (magnification ×200, scale bar: 50 μm). *p < 0.05, **p < 0.01.

To elucidate and understand whether dysbiosis of gut microbiota is causal for colonic mucosal barrier defects, we analyzed mucosal barrier function in recipient mice. Mice colonized with the CRS microbiota exhibited disrupted colonic mucosal barrier before and after DSS exposure, while *A. muciniphila* supplementation significantly enhanced the expression of MUC2 (**Figure 8A**) and increased the number of goblet cells and MUC2-positive cells in each villus (**Figures 8B** **–E**).

#### *A. muciniphila* Supplementation Remodeled the Gut Microbiota in Recipient Mice

**Figure 9:**
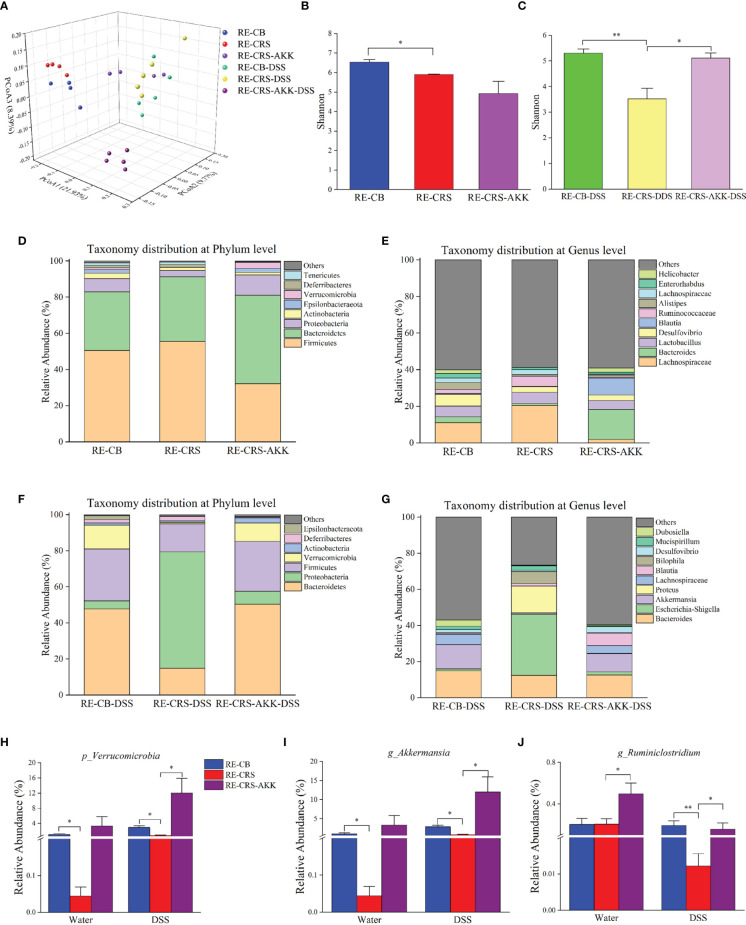
*A. muciniphila* supplementation remodeled the gut microbiota in recipient mice. **(A)** PCoA according to unweighted Unifrac revealed site-specific clustering (n = 4–5). **(B, C)** The alpha diversity, Shannon index were shown in each recipient group; **(D–G)** relative abundance compositions of microbiota at the phylum and genus level presented in each recipient group. **(H)** The representative phylum of *Verrucomicrobia* was differentially represented after *A. muciniphila* treatment in recipient mice. **(I–J)** Two representative genera were differentially represented after *A. muciniphila*. *p < 0.05, **p < 0.01.

Based on the 16S rRNA gene-based profile, the Shannon diversity index revealed that *A. muciniphila* supplementation increased alpha diversity after DSS exposure compared to recipient mice colonized with the CRS microbiota (**Figure 9C**). Moreover, *A. muciniphila* supplementation revealed no significant difference in the Shannon diversity index before and after DSS administration compared with recipient mice colonized with the CB microbiota, indicating that alpha diversity was partly recovered after *A. muciniphila* treatment (**Figures 9B, C**). PCoA on unweighted UniFrac distances was performed to characterize differences among these groups (**Figure 9A**). ANOSIM analysis showed that *A. muciniphila* supplementation resulted in distinct gut microbial communities before and after DSS administration compared with the mice colonized with the CRS microbiota (RE-CB with RE-CRS-AKK: R = 0.82, p = 0.03; RE-CB-DSS with RE-CRS-AKK-DSS: R = 0.89, p = 0.01). The microbial composition at the phylum and genus levels in each recipient group is shown in **Figures 9D** **–G**. In agreement with Experiment 1, the relative abundance of *Verrucomicrobia* and *Akkermansia* was decreased in mice colonized with the CRS microbiota before and after DSS administration than in mice colonized with the CB microbiota. Following DSS exposure, *A. muciniphila* supplementation significantly increased the relative abundance of *Verrucomicrobia* and *Akkermansia* (**Figures 9H, I**). Notably, *A. muciniphila* supplementation significantly enhanced the relative abundance of *Ruminiclostridium* (**Figure 9J**).

## Discussion

A growing body of evidence has revealed that depression has negative effects on the course of IBD, but the influence of these psychological disorders on IBD severity remains obscure. In this study, we demonstrated that CRS aggravates DSS-induced colitis with shorter colon length, higher histopathological scores, colonic mucus damage, and gut microbiota dysbiosis. The associations between psychological disorders and IBD through the brain-gut axis, including the hypothalamus-pituitary axis, activation of the sympathetic nervous system, increased proinflammatory cytokines, and decreased intestinal permeability (Bonaz and Bernstein, 2013; Brzozowski et al., 2016; Labanski et al., 2020). However, the exact mechanisms underlying the interaction between gut microbiota and colonic mucus require further exploration.

In the last decade, the effects of the gut microbiota on IBD have received much attention. It is widely reported that gut microbiota disturbance or dysbiosis influences the progress of IBD (Ananthakrishnan, 2015; Ananthakrishnan et al., 2018). Numerous studies from animal models and clinical studies have indicated that stress clearly causes gut microbiota dysbiosis (Galley and Bailey, 2014; Bharwani et al., 2016; Osadchiy et al., 2019). In this study, we also found that dysbiosis induced by CRS is characterized by a dramatic alteration in the gut microbiota construction and composition. Notably, a reduced abundance of *A. muciniphila* was observed in mice subjected to CRS. A large-scale microbiome population cohort from patients with depression demonstrated that *A. muciniphila* could promote the secretion of serotonin, which indicated that *A. muciniphila* plays an important role in depression (Valles-Colomer et al., 2019). In our study, we found that *A. muciniphila* was reduced in UC patients with depression. This discovery has great potential for the search of new biomarkers and treatment for IBD with psychological disorders.

IBD is also associated with damage of colonic mucosal barriers, or so-called “leaky gut.” The colonic mucus largely relies on the release of MUC2. A hydrated glycosylated protein, MUC2, is produced by goblet cells, adhering to the surface of the colon to prevent luminal microbes and pathogen invasion (Hansson and Johansson, 2010; Johansson et al., 2011). Furthermore, multiple studies have indicated that colonic barrier loss occurs in mice under stress conditions (Söderholm et al., 2002; Yu et al., 2010; Oliver et al., 2012). Although our methods were different, we also observed a decrease in MUC2-positive cells and goblet cells in each villus after 30 days of CRS. The mRNA expression of MUC2 in colon tissue was also significantly decreased in mice under CRS. Previous studies have shown that MUC2^−/−^ mice exhibit severe colitis, which is accompanied by a remarkable downregulation of goblet cells (Morampudi et al., 2016). These results suggest that CRS-associated colonic mucus defects may lead to the development of colitis.

Culminating from the changes of gut microbiota paralleled the damage of colonic mucus in Experiment 1, we aim to determine whether gut microbiota is causal for the colonic mucosal defects. Therefore, we designed an experiment using a gut microbiota-depletion mouse model followed by engraftment by either CRS mice or control CB donor mice to indicate the critical role of gut microbiota in pathogenesis. In Experiment 2, we observed that recipient mice with transplantation of CRS microbiota exhibited severe colitis. Meanwhile, reduced numbers of MUC2-positive cells and goblet cells, together with a reduction of MUC2 mRNA levels, were observed in the colons of recipient mice colonized with the CRS microbiota, indicating that CRS-mediated gut microbiota changes are responsible for colonic mucus damage and colitis aggravation. Interestingly, depression-like symptoms were observed in recipient mice colonized with microbiota obtained from CRS mice. This finding supports the potential that the “brain-gut-microbiota” axis may exist as a bidirectional interaction and communication (Rhee et al., 2009; Al Omran and Aziz, 2014).

16S rRNA high-throughput sequencing showed that the microbiota composition of the CRS donor and recipient mice was reduced in *A. muciniphila*. Furthermore, MUC2 levels were positively correlated with the abundance of *A. muciniphila*. Considering this relationship, we investigated whether *A. muciniphila* is causally involved in colonic mucus repair. Consistently, *A. muciniphila* supplementation prevented colitis aggravation and strengthened colonic mucosal barriers in recipient mice by improving the expression of MUC2 and increased the number of goblet cells and MUC2-positive cells. *A. muciniphila* has been identified as a probiotic and has many reported beneficial effects, including promoting the metabolism of the mucus layer, thereby promoting a healthy microenvironment of epithelial cells and gut barrier integrity (Derrien et al., 2004; Derrien et al., 2010; Everard et al., 2013). Moreover, *A. muciniphila* has been found to restore a damaged gut barrier *in vitro* (Reunanen et al., 2015). Our findings are in agreement with previous results.

Our previous studies demonstrated that FMT or probiotic therapy was identified as novel interventions for gastrointestinal disease, which is attributed to the normalization of gut microbial dysbiosis (Chen et al., 2018; Bu et al., 2020). The present study indicated that *A. muciniphila* supplementation increased the relative abundance of *Ruminiclostridium* in recipient mice. *Ruminiclostridium* is an anaerobic bacterium that degrades cellulose (Xu et al., 2013; Xu et al., 2015). A recent study has shown that the levels of *Ruminiclostridium* correlate with an increased number of microglia, suggesting that *Ruminiclostridium* plays an important role in the brain-gut axis (Loman et al., 2019). These results revealed that *A. muciniphila* not only prevented damage to colonic mucus but also modified gut microbiota.

This study has certain limitations. First, *A. muciniphila* supplementation alleviated depression-like behaviors in recipient mice before DSS administration. Accordingly, we detected the colonization of *A. muciniphila* by 16S rRNA sequencing. The relative abundance of *A. muciniphila* supplementation tended to increase, but no statistically significant difference was observed. The failure of colonization suggested that *A. muciniphila* should undergo repeated transfers, probably due to its sensitivity to oxygen (Derrien et al., 2004). Further studies on the mechanisms of *A. muciniphila* on antidepressant phenotypes are needed. Additionally, *A. muciniphila* inhibits the aggravation of colitis in mice under CRS, and clinical studies on the efficacy and safety of *A. muciniphila* in IBD patients with depression are warranted.

## Conclusion

This study demonstrated that CRS-mediated gut microbiota dysbiosis resulted in damage to colonic mucus and the development of colitis. *A. muciniphila* supplementation protected colonic mucus and inhibited aggravation of colitis. Our findings may open new probiotic options for treating IBD patients with psychological disorders, which are involved in the damage of colonic mucus.

## Data Availability Statement

The datasets presented in this study can be found in online repositories. The names of the repository/repositories and accession number(s) can be found below: https://www.ncbi.nlm.nih.gov/, PRJNA756202 and PRJNA758283.

## Ethics Statement

The studies involving human participants were reviewed and approved by the Institutional Review Board of Jiangsu Provincial Hospital of Traditional Chinese Medicine. The patients/participants provided their written informed consent to participate in this study. The animal study was reviewed and approved by the Ethics Committee for Animal Experiments of Jiangsu Provincial Hospital of Traditional Chinese Medicine. Written informed consent was obtained from the individual(s) for the publication of any potentially identifiable images or data included in this article.

## Author Contributions

TC and RW designed the experiments and wrote the original manuscript. ZD, XY, YD, ZF, and FB performed the experiments. QW, JZ, and LL analyzed the data. YC, GS, and QN edited the original manuscript. All of authors contributed to the article and approved the submitted version.

## Funding

This study was supported by the Priority Academic Program Development of Jiangsu Higher Education Institutions and the leading talent project of Chinese medicine of Jiangsu Province (Grant No. SLJ0203).

## Conflict of Interest

The authors declare that the research was conducted in the absence of any commercial or financial relationships that could be construed as a potential conflict of interest.

## Publisher’s Note

All claims expressed in this article are solely those of the authors and do not necessarily represent those of their affiliated organizations, or those of the publisher, the editors and the reviewers. Any product that may be evaluated in this article, or claim that may be made by its manufacturer, is not guaranteed or endorsed by the publisher.
