# Supplementary material for: Akkermansia muciniphila Protects Against Psychological Disorder-Induced Gut Microbiota-Mediated Colonic Mucosal Barrier Damage and Aggravation of Colitis
Source: Front Cell Infect Microbiol. 2021 Oct 14;11:723856. doi: 10.3389/fcimb.2021.723856 (PMC8551916; doi:10.3389/fcimb.2021.723856)
Supplement: Supplementary file 1 [file Table_1.docx]

Supplementary Material


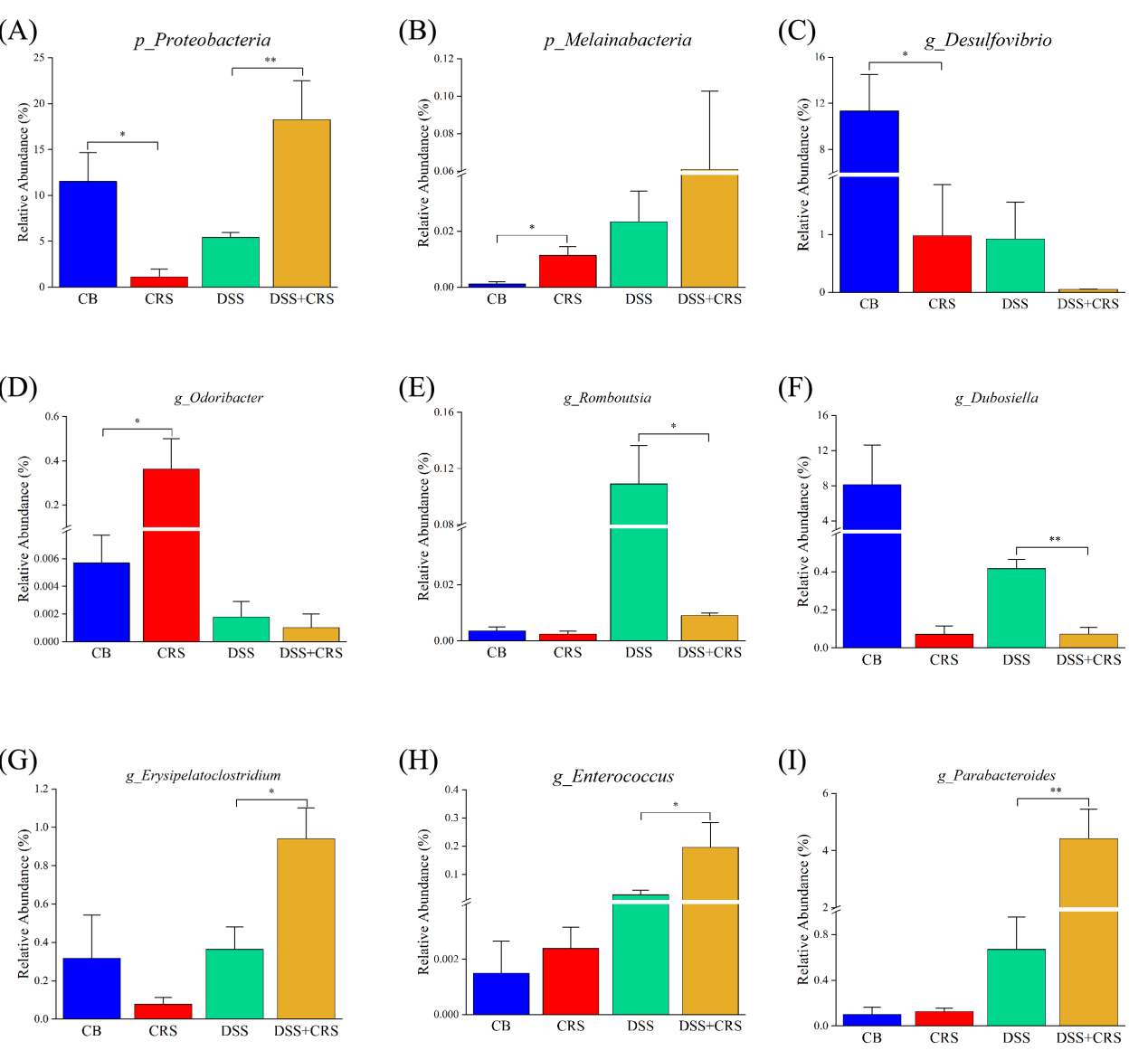


**Supplementary Figure 1.** Gut microbial profile analysis were conducted from the comparisons of CB *versus* CRS, and DSS *versus* DSS + CRS at the phylum and genus level.


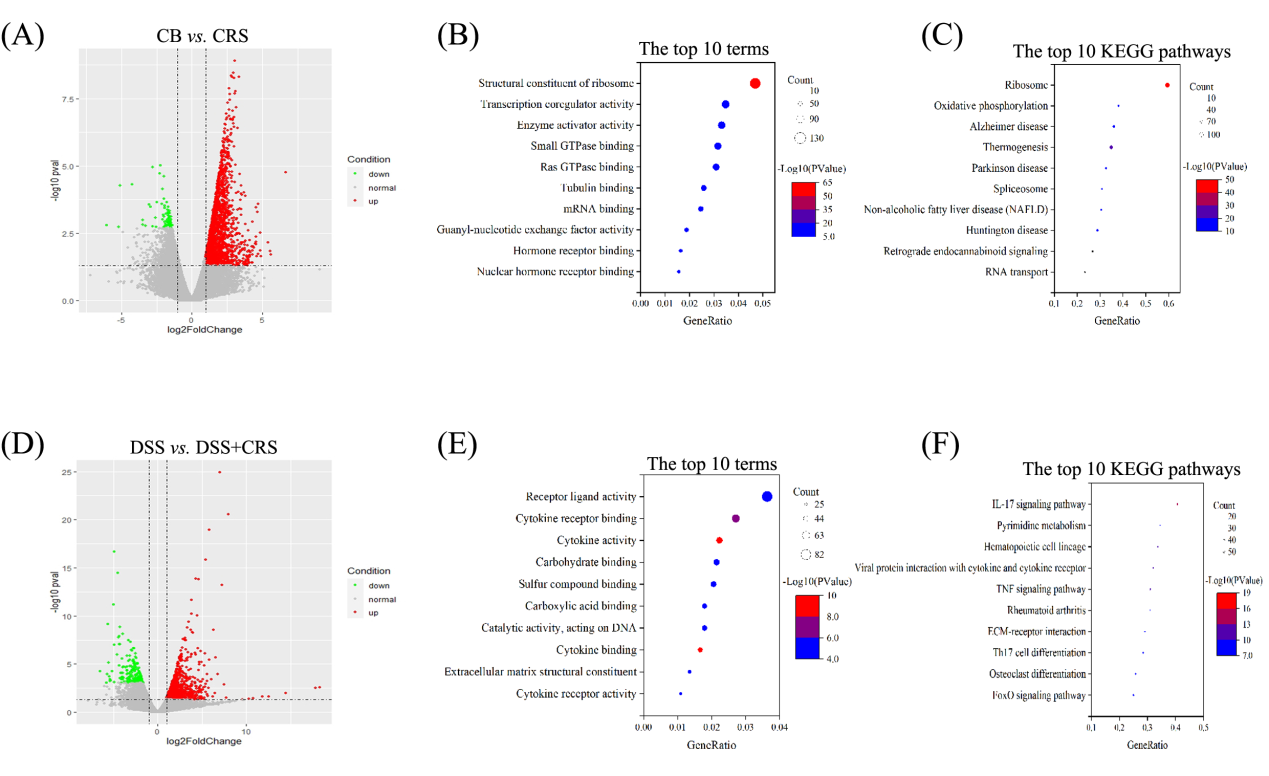


**Supplementary Figure 2.** Volcano plot shown DEGS from the comparisons of CB *versus* CRS (A), and DSS *versus* DSS + CRS (D); The DEGS between CB and CRS were analyzed by GO (B) and KEGG pathway analysis (C); The DEGS between DSS and DSS+CRS were analyzed by GO (E) and KEGG pathway analysis (F).

**Supplementary Table 1.** Patients characteristics

|  | UC (N = 19) | UC with depression (N = 16) | *p* value^b^ |
| --- | --- | --- | --- |
| Female gender (%) | 9 (47.37) | 9 (56.25) | 0.51 |
| Age (years) ^a^ | 43.68 ± 2.92 | 41.31 ± 3.05 | 0.58 |
| BMI^a^ | 21.08 ± 0.79 | 22.49 ± 0.66 | 0.19 |
| Smoker (%) | 5 (26.32) | 6 (37.50) | <0.001 |
| PHQ-9 score^a^ | 2.26 ± 0.31 | 13.75 ± 0.99 | <0.001 |

^a^Mean ± SEM;

^b^Independent samples ANOVA for continuous data and χ^2^ for categorical data.

**Supplementary Table 2.** Primers applied in the qPCR assays.

| Gene | Primer Sequences(5’-3’) |
| --- | --- |
| **MUC2** |  |
| Forward | TGCCCACCTCCTCAAAGAC |
| Reverse | TAGTTTCCGTTGGAACAGTGAA |
| **GAPDH** |  |
| Forward | AGAACATCATCCCTGCATCC |
| Reverse | CTGGGATGGAAATTGTGAGG |

**Supplementary Table 3.** Top 15 up-regulated genes on colonic tissue of mice from the comparisons of CB *versus* CRS, and DSS *versus* DSS+CRS.

| **Gene ID** | **Gene name** | **log2FoldChange** | **p-Value** |
| --- | --- | --- | --- |
| **CRS *vs.* CB** | | | |
| KRT83 | keratin 83 | 5.434076 | 0.006839 |
| IL22 | interleukin 22 | 4.05741 | 0.040013 |
| S100A8 | S100 calcium binding protein A8 | 4.014502 | 0.002449 |
| LY6G | lymphocyte antigen 6 complex, locus G | 3.588409 | 0.006813 |
| S100A9 | S100 calcium binding protein A9 | 3.403554 | 0.010517 |
| CXCL1 | C-X-C motif chemokine ligand 1 | 3.305717 | 0.00315 |
| SNHG3 | small nucleolar RNA host gene 3 | 2.956967 | 0.001274 |
| CXCL5 | C-X-C motif chemokine ligand 5 | 2.617889 | 0.008312 |
| MYH4 | myosin heavy chain 4 | 2.577951 | 0.00054 |
| TMEM40 | transmembrane protein 40 | 2.472826 | 0.022501 |
| SAA2 | serum amyloid A2 | 2.447801 | 0.01012 |
| HOTAIRM1 | HOXA transcript antisense RNA, myeloid-specific 1 | 2.241401 | 0.00451 |
| CEBPD | CCAAT enhancer binding protein delta | 1.876679 | 0.000368 |
| PABPC1L | poly(A) binding protein cytoplasmic 1 like | 1.841038 | 0.038295 |
| CNBD2 | cyclic nucleotide binding domain containing 2 | 1.750191 | 0.00034 |
| **DSS+CRS *vs.* DSS** | | | |
| CXCL1 | C-X-C motif chemokine ligand 1 | 3.853442 | 4.78E-09 |
| MT2 | metallothionein 2 | 3.741504 | 1.73E-06 |
| LY6G | lymphocyte antigen 6 complex, locus G | 3.119192 | 0.004794609 |
| KRT83 | keratin 83 | 2.853133 | 0.010173141 |
| S100A8 | S100 calcium binding protein A8 | 2.736632 | 0.001259154 |
| S100A9 | S100 calcium binding protein A9 | 2.700637 | 0.003915279 |
| CTLA2A | cytotoxic T lymphocyte-associated protein 2 alpha | 2.631445 | 0.004790984 |
| TMEM40 | transmembrane protein 40 | 2.562537 | 0.001855793 |
| MYH4 | myosin heavy chain 4 | 2.535989 | 0.025817432 |
| SAA2 | serum amyloid A2 | 2.417781 | 0.007571333 |
| IL22 | interleukin 22 | 2.26722 | 0.004686736 |
| CXCL5 | C-X-C motif chemokine ligand 5 | 2.206451 | 3.87E-05 |
| PABPC1L | poly(A) binding protein cytoplasmic 1 like | 2.204003 | 0.013783805 |
| HOTAIRM1 | HOXA transcript antisense RNA, myeloid-specific 1 | 1.859712 | 0.010426631 |
| HILPDA | hypoxia inducible lipid droplet associated | 1.604826 | 0.001495216 |

**Supplementary Table 4.** Top 15 down-regulated genes on colonic tissue of mice from the comparisons of CB *versus* CRS, and DSS *versus* DSS+CRS.

| **Gene ID** | **Gene name** | **log2FoldChange** | **p-Value** |
| --- | --- | --- | --- |
| **CRS *vs.* CB** | | | |
| ANO7 | anoctamin 7 | -2.76602 | 1.12E-05 |
| SYT15 | synaptotagmin 15 | -2.36986 | 0.003714 |
| CX3CR1 | C-X3-C motif chemokine receptor 1 | -1.9698 | 0.026449 |
| CBFA2T3 | CBFA2/RUNX1 partner transcriptional co-repressor 3 | -1.88644 | 0.003505 |
| KIF14 | kinesin family member 14 | -1.86305 | 0.036584 |
| SPIB | Spi-B transcription factor | -1.65594 | 0.049013 |
| NCAPD2 | non-SMC condensin I complex subunit D2 | -1.64815 | 0.004666 |
| FANCD2 | FA complementation group D2 | -1.64512 | 0.014777 |
| TENM4 | teneurin transmembrane protein 4 | -1.61736 | 0.04904 |
| MYO7B | myosin VIIB | -1.56633 | 0.010165 |
| TMEM94 | transmembrane protein 94 | -1.54699 | 0.000571 |
| CLSPN | claspin | -1.52145 | 0.024285 |
| CMAH | cytidine monophospho-N-acetylneuraminic acid hydroxylase | -1.45743 | 0.002198 |
| B4GALNT2 | beta-1,4-N-acetyl-galactosaminyltransferase 2 | -1.4371 | 0.003287 |
| MUC2 | mucin 2 | -1.41677 | 0.007726 |
| **DSS+CRS *vs.* DSS** | | | |
| RTN4R | reticulon 4 receptor | -3.91331 | 0.00041 |
| SYT15 | synaptotagmin 15 | -3.4449 | 0.001795 |
| CX3CR1 | C-X3-C motif chemokine receptor 1 | -3.39139 | 0.000292 |
| KNTC1 | kinetochore associated 1 | -2.4288 | 0.007091 |
| TENM4 | teneurin transmembrane protein 4 | -2.3386 | 0.01926 |
| FANCD2 | FA complementation group D2 | -2.10129 | 0.023573 |
| MUC2 | mucin 2 | -2.0953 | 0.019451 |
| ANO7 | anoctamin 7 | -2.06656 | 0.017739 |
| INTS3 | integrator complex subunit 3 | -2.00921 | 0.00077 |
| ZBTB40 | zinc finger and BTB domain containing 40 | -2.0037 | 0.01368 |
| TOP2A | DNA topoisomerase II alpha | -2.00326 | 0.000974 |
| B4GALNT2 | beta-1,4-N-acetyl-galactosaminyltransferase 2 | -1.94606 | 0.000263 |
| CLSPN | claspin | -1.91863 | 0.017774 |
| TICRR | TOPBP1 interacting checkpoint and replication regulator | -1.90788 | 0.03053 |
| KIF14 | kinesin family member 14 | -1.89234 | 0.038941 |
